# Strong Ion Regulatory Abilities Enable the Crab *Xenograpsus testudinatus* to Inhabit Highly Acidified Marine Vent Systems

**DOI:** 10.3389/fphys.2016.00014

**Published:** 2016-02-01

**Authors:** Marian Y. Hu, Ying-Jey Guh, Yi-Ta Shao, Pou-Long Kuan, Guan-Lin Chen, Jay-Ron Lee, Ming-Shiou Jeng, Yung-Che Tseng

**Affiliations:** ^1^Institute of Cellular and Organismic Biology, Academia SinicaTaipei, Taiwan; ^2^Institute of Physiology, Christian-Albrechts University KielKiel, Germany; ^3^Institute of Biological Chemistry, Academia SinicaTaipei, Taiwan; ^4^Institute of Marine Biology, National Taiwan Ocean UniversityKeelung, Taiwan; ^5^Department of Life Science, National Taiwan Normal UniversityTaipei, Taiwan; ^6^Biodiversity Research Center, Academia SinicaTaipei, Taiwan

**Keywords:** hydrothermal vent, V-type H^+^-ATPase, Na^+^/K^+^-ATPase, hypercapnia, invertebrate physiology, gill, crustacean

## Abstract

Hydrothermal vent organisms have evolved physiological adaptations to cope with extreme abiotic conditions including temperature and pH. To date, acid-base regulatory abilities of vent organisms are poorly investigated, although this physiological feature is essential for survival in low pH environments. We report the acid-base regulatory mechanisms of a hydrothermal vent crab, *Xenograpsus testudinatus*, endemic to highly acidic shallow-water vent habitats with average environment pH-values ranging between 5.4 and 6.6. Within a few hours, *X. testudinatus* restores extracellular pH (pHe) in response to environmental acidification of pH 6.5 (1.78 kPa pCO_2_) accompanied by an increase in blood ${\text{HCO}}_{3}^{-}$ levels from 8.8 ± 0.3 to 31 ± 6 mM. Branchial Na^+^/K^+^-ATPase (NKA) and V-type H^+^-ATPase (VHA), the major ion pumps involved in branchial acid-base regulation, showed dynamic increases in response to acidified conditions on the mRNA, protein and activity level. Immunohistochemical analyses demonstrate the presence of NKA in basolateral membranes, whereas the VHA is predominantly localized in cytoplasmic vesicles of branchial epithelial- and pillar-cells. *X. testudinatus* is closely related to other strong osmo-regulating brachyurans, which is also reflected in the phylogeny of the NKA. Accordingly, our results suggest that the evolution of strong ion regulatory abilities in brachyuran crabs that allowed the occupation of ecological niches in euryhaline, freshwater, and terrestrial habitats are probably also linked to substantial acid-base regulatory abilities. This physiological trait allowed *X. testudinatus* to successfully inhabit one of the world's most acidic marine environments.

## Introduction

**Figure 1:**
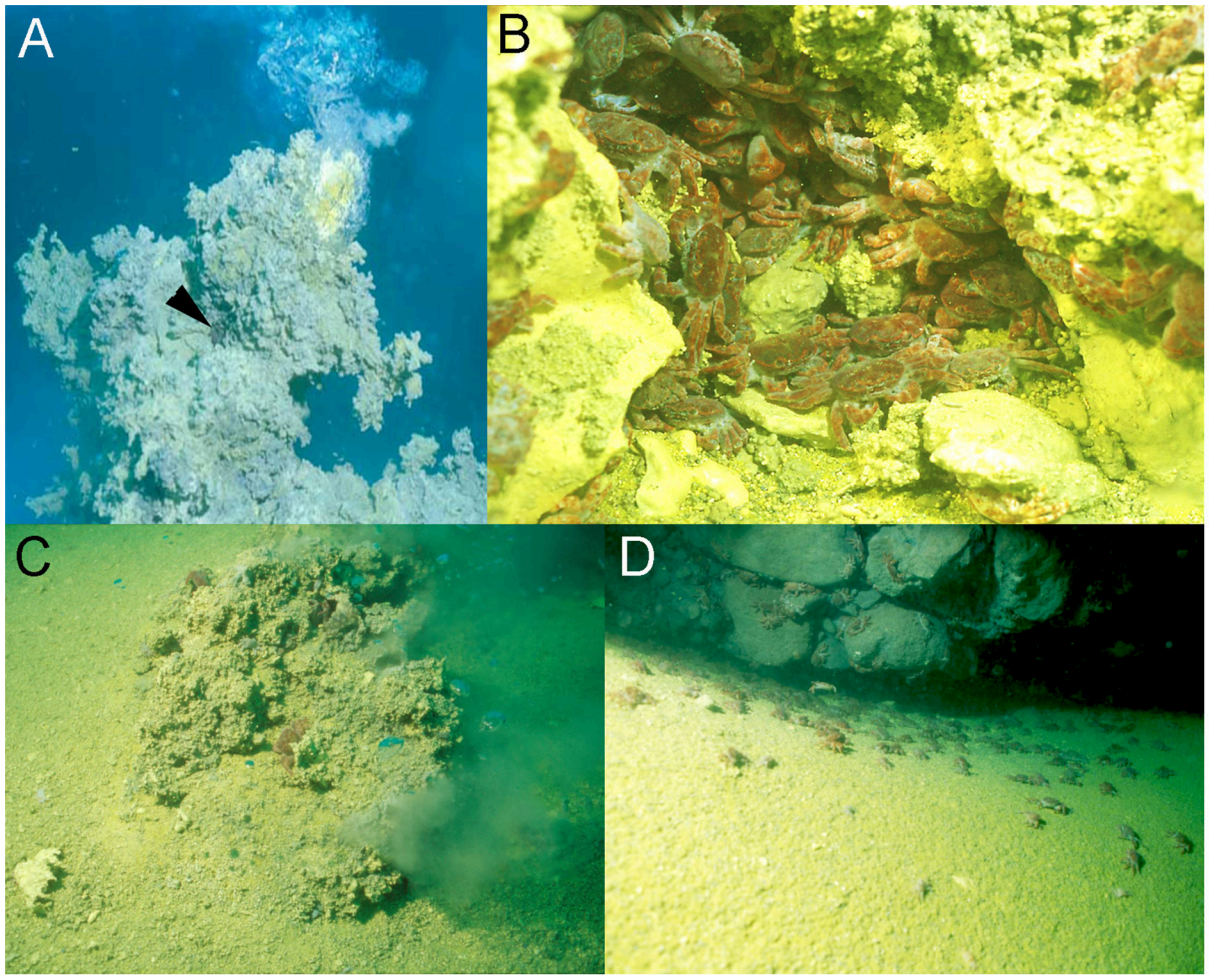
*****Xenograpsus testudinats*** crabs in the hydrothermal vent habitat at Kueishan island located off Taiwan's east coast**. Underwater photographs of the hydrothermal vent system at Kueishan island, showing an active chimney discharging acidic, CO_2_ rich water (note the *X. testudinatus* crab in the direct vicinity of the vent opening) **(A)**. High densities of *X. testudinatus* crabs can be found in the direct surrounding of the vents hiding in sulfur-rich crevices **(B)**. Whilst feeding on zooplankton killed by the toxic discharges of the vents crabs swarm out of their shelters, and are directly exposed to highly acidic waters **(C,D)**. All images were obtained and authorized from the co-author Dr. Ming-Shiou Jeng.

Deep sea hydrothermal vent systems support ecosystems with an enormous biomass, and reveal a rich biodiversity ranging from microbes to vertebrates (Tunnicliffe, 1992). To survive in these extreme habitats, vent associated organisms show a range of morphological and physiological adaptations to cope with challenging environmental conditions including temperature, metallic sulfides, anoxia, hypercapnia, and low pH (Goffredi et al., 1997; Ramirez-Llodra et al., 2007). Highly acidified conditions due to the release of HCl and CO_2_ are a characteristic of most seafloor vent systems including the shallow-water hydrothermal vent system of Kueishan Island (24°50′N, 121°57′E), off the coast of Taiwan (Han et al., 2014). This shallow water hydrothermal vent system has been described as one of the most acidic vents in the world, discharging water with a high content of elemental sulfur particles, having temperatures ranging between 76 and 116°C and a minimum pH of 1.52 (Chen et al., 2005; Figure 1A). The gas composition released by the underwater volcano is mainly CO_2_ (<92%; Han et al., 2014). Even in the surrounding areas with depths between 2 and 14 m, the seawater is highly acidic ranging from pH 6.6 to 5.4 (Han et al., 2014). This challenging hydrothermal vent habitat is inhabited by *Xenograpsus testudinatus*, a crab species that is endemic to shallow-water (<200 m) vent systems (Ng et al., 2000). *X. testudinatus* is the only metazoan species found in the direct surroundings of the vents, and individuals congregate in large numbers in vent crevices with an average of 364 individuals per m^2^ (Figure 1B). These crabs have evolved a unique feeding behavior by feeding on dead zooplankton killed by the toxic vent discharges (Jeng et al., 2004). During slack water conditions, when there are no currents, the crabs swarm out of their crevices (Figures 1C,D) to rapidly feed on this “marine snow” of dead zooplankton (Jeng et al., 2004).

Crustaceans are probably one of the most successful invertebrate group that occupy ecological niches in marine systems ranging from polar regions (Frederich et al., 2001) to hydrothermal vents (Joel and Haney, 2005; Dittel et al., 2008) and have radiated from seawater to freshwater and even into terrestrial habitats (Schubart et al., 1997). While those found in stenohaline marine habitats are more likely to be weak osmo-regulators or even osmo-conformers crustaceans from euryhaline or intertidal habitats are moderate to strong osmo-regulators, a physiological feature that is beneficial for adaptation to habitats with fluctuating salinities (Charmantier and Charmantier-Daures, 2001; Henry et al., 2012). The gills are the major organ involved in extracellular ion homeostasis, equipped with an efficient ion regulatory machinery that shows evolutionary conserved features comparable to the ion regulatory epithelia in cephalopods and fish (Henry et al., 2012). Similar to the situation in most vertebrate and invertebrate systems, the ubiquitous Na^+^/K^+^-ATPase localized in basolateral membranes creates the electro-chemical gradient that is used by secondary active transporters such as apical Na^+^/H^+^-exchangers or anion exchangers that can mediate ionic and pH homeostasis in crustaceans (Cameron, 1978; Heisler, 1986; Pörtner et al., 1991; Gutowska et al., 2010; Henry et al., 2012). The V-type H^+^-ATPase (VHA) has also been described in gill epithelia of crustaceans, demonstrating a cytosolic or apical localization of this enzyme (Weihrauch et al., 2001; Tsai and Lin, 2007). The VHA has been shown to be involved in the acidification of intracellular organelles, and in the secretion of protons across the plasma membrane of specialized cells located in ion-regulatory epithelia (Weihrauch et al., 2002; Tresguerres et al., 2006; Hwang, 2009; Hu et al., 2014). Based on the structure of the VHA with the catalytic (V1 complex) site facing the cytoplasm, the VHA is restricted to transport protons out of the cytoplasm (Beyenbach, 2006).

In euryhaline crabs changes in environmental salinity were demonstrated to directly affect the extracellular acid-base status of these animals (Truchot, 1981, 1992). The fact that a reduction in environmental salinity induces a metabolic alkalosis while an increase in salinity leads to a metabolic acidosis demonstrated that osmo-regulation and the maintenance of acid-base homeostasis are directly linked (Whiteley et al., 2001). This connection between osmo and acid-base regulation may explain why most osmo-regulating crustaceans are relatively tolerant to environmental acid-base disturbances (Henry and Cameron, 1982; Spicer et al., 2007).

*X. testudinatus* is a brachyuran crab species that has a phylogenetic position close to other crab species that are characterized as strong osmo-regulators including the euryhaline crab *Eriocheir sinensis* and crabs of the genus *Hemigrapsus* spp. (Hicks, 1973; Bedford and Leader, 1977; Onken and Graszynski, 1989; Ki et al., 2009). The potential link between acid-base and osmo-regulation in many euryhaline decapod crustaceans prompted us to formulate the hypothesis that *X. testudinatus* utilizes conserved ion pumps to regulate extracellular pH. To test this hypothesis we exposed *X. testudinatus* to CO_2_-induced seawater acidification (pH 6.5), and monitored changes in extracellular acid-base status as well as expression, protein concentrations and activities of the branchial Na^+^/K^+^-ATPase and V-type H^+^-ATPase over a time course of 48 h. These results will demonstrate that evolution of strong osmo-regulatory abilities in this phylogenetic group may represent a requisite that is accompanied by substantial acid-base regulatory abilities that allowed *X. testudinatus* to inhabit one of the most acidic marine habitats.

## Materials and methods

### Acidification experiments

*X. testudinatus* with carapace width ranging between 3 to 4 cm were obtained by SCUBA divers from Kueishan Island, Taiwan (ROC) in July 2013. Crabs were collected from depth ranging between 5 and 10 m and held in recirculating natural seawater systems (500 l total volume, nitrification filter, salinity 31–32‰, temperature 28°C, constant 12 h dark:12 h light cycle) at the Institute of Cellular and Organismic Biology, Academia Sinica. Animals were fed *ad libitum* twice per day with tilapia meat. CO_2_ perturbation experiments were carried our using a total of 48 animals that were distributed into six 20 l tanks (8 animals per tank). The six tanks, with three replicate tanks for each pH treatment were connected to a flow through system providing filtered (0.2 μm), natural seawater. Water was exchanged at a flow rate of ~3 l h^−1^ to guarantee high water quality inside the test aquaria. The pH (8.0 and 6.5) in the experimental tanks was continuously adjusted by the addition of the appropriate gas mixtures using a continuous pH-stat system (pH controller, MACRO) that controlled the addition of pure CO_2_ into the seawater. The experimental tanks were additionally aerated with air (O_2_ saturation > 90%) to assure sufficient seawater pO_2_ during the experiment. Specific seawater physicochemical conditions for the different incubations are shown in Table S1. Animals were not fed during the 48 h experimental period in order to minimize physiological artifacts caused by feeding activity. pH_SW_ was measured using a WTW 340i meter equipped with a WTW SenTix 81 electrode that was calibrated daily with Tris and AMP buffers at a salinity of 31 to monitor the experiment. Alkalinity in seawater samples was determined spectrophotometrically according to Sarazin et al. (1999). The carbonate system of seawater was calculated from A_T_ and pH_SW_ using the software CO2SYS (Lewis and Wallace, 1998), including the dissociation constants of Mehrbach et al. (1973) as refitted by Dickson and Millero (1987). Along the experimental duration of 48 h, extracellular acid-base parameters were measured, and gill tissue samples were collected for gene expression, protein, and enzyme analyses. Tissue and hemolymph sampling was carried out at six time points (0, 1, 4, 12, 24, and 48 h) along the time course of 48 h. At each sampling time point, one to two animals were sampled from the three experimental replicate tanks, leading to a biological replication of *n* = 4. Animals were anesthetized by cooling on ice and were killed by an incision of the frontal region of the carapace. The carapace was removed to access the branchial chamber where the gills are located. *X. testudinatus* has six gill pairs (Figures S2A,B), of which the anterior-most gill 1 (G1), is minute (length 1–2 mm); G6 is the posterior-most gill with a total length of ~10 mm. G1–G3 are designated as anterior gills, while G4–6 are considered posterior gills. G3–G6 are similar in size while G2 is smaller (~5 mm long). The gill formula of *X. testudinatus* is: G1,G2 podobranch, G3 and G4 arthrobranchs with a common insertion point, G5 and G6 arthrobranchs with a common insertion point. Gills from the left side were sampled for gene expression studies while those from the right side were sampled for protein and enzyme analyses. Pooled samples of posterior gills (4,5,6) were used for gene expression, activity and protein analyses. The experimental protocols for the present study were approved by the National Taiwan Normal University Institutional Animal Care and Utilization Committee (approval no.: 101005).

### Extracellular acid-base status

Hemolymph samples were taken from the coxa using a gas-tight Hamilton syringe. Determination of pH_e_ in venous hemolymph was performed in 100 μl samples using a microelectrode (WTW Mic-D) and a WTW 340 pH meter (precision ± 0.01 units) that was calibrated with Radiometer precision buffers 7 and 10 (S11M44, S11 M007). Measurements were performed inside a temperature controlled water bath adjusted to 28°C. Due to low hemolymph sample volumes for total dissolved inorganic carbon (*C*_T_) determinations (~100 μl) samples were diluted 1:1 with de-ionized water prior to measurements. After hemolymph sampling, the dilution and measurement of samples was carried out within less than 30 s to guarantee negligible changes in the carbonate system of hemolymph samples. *C*_T_ was determined in duplicates (100 μl each) via a Corning 965 carbon dioxide analyzer (precision ± 0.1 mmol l^−1^; Olympic Analytical Service, England) that was calibrated using a fresh dilution series of 40, 20, 10, 5, and 2.5 mM bicarbonate in distilled water to generate a sodium bicarbonate standard curve. Carbonate system speciation (i.e., *p*CO_2_, [${\text{HCO}}_{3}^{-}$]) of hemolymph samples of *X. testudinatus* was calculated from extracellular pH (pH_e_) and *C*_T_ using the Henderson–Hasselbalch equation with dissociation constants and solubility coefficients as previously described for the shore crab *Carcinus maenas* (Truchot, 1976).

### Immunohistochemistry and western blot analyses

For immunohistochemistry gill tissues from control animals were fixed and mounted to slides as previously described (Hu et al., 2014). The primary antibodies, a mouse monoclonal antibody α5, raised against the avian α subunit of the Na^+^/K^+^-ATPase (Hybridoma Bank) and a polyclonal antibody raised against part the subunit A region (SYSKYTRALDEFYDK) of the molluscan V-type-H^+^-ATPase (VHA; for more detail see Hu et al., 2013) were diluted in PBS (1:100) and placed in droplets of 200 μl onto the sections, and incubated over night at 4°C inside a wet chamber. Sections were then washed (3 × 5 min) with PBS and incubated for 1 h with the secondary antibody, anti-mouse Alexa Fluor 488, or anti-rabbit Alexa Fluor 568 (Invitrogen) (dilution 1:250). After rinses in PBS (3 × 5 min), sections were examined and photographed using a fluorescence microscope (Zeiss imager A1) equipped with an appropriate filter set. Negative controls were performed several times for every antibody by omitting the primary antibody.

Immunoblotting was essentially performed as previously described (Hu et al., 2014) using 15 μL of gill crude extracts. Proteins were fractionated by SDS-PAGE on 10% polyacrylamide gels, and transferred to PVDF membranes (Millipore), using a tank blotting system (Bio-Rad). Blots were exposed to the primary antibody (see previous section) diluted 1:250–500 and incubated at 4°C overnight. After washing with PBS-T (phosphate buffered saline containing 0.1% Tween20), blots were incubated for 2 h with horseradish conjugated goat anti-rabbit IgG antibody (diluted 1:1000–2000, at room temperature; Amersham Pharmacia Biotech). Protein signals were visualized using the enhanced chemiluminescence system (ECL, Amersham Pharmacia Biotech) and recorded using Biospectrum 600 imaging system (UVP, Upland, CA, USA). Signal intensities were calculated using the free software “Image J” (e.g., Schneider et al., 2012).

### Enzyme activity

ATPase activity was measured in crude extracts of the three posterior gills (4,5,6). The measurement is based on a coupled enzyme assay containing pyruvate kinase (PK) and lactate dehydrogenase (LDH) as previously described (Hu et al., 2014). Crude extracts were obtained by quickly homogenizing the tissue samples using a pestle followed by complete homogenization in a tissue lyzer (Qiagen) in five volumes of ice-cold imidazole buffer (Hu et al., 2014). After centrifugation for 10 min at 1000 g and 4°C, cell debris was removed and the supernatant was used as a crude extract. The reaction was started by adding 1.5 μl of the sample homogenate to the reaction buffer. The coupled to The hydrolysis of ATP reflected by the oxidation of NADH was measured photometrically at 30°C in a temperature controlled plate reader (Molecular Device, Spectra Max, M5), over a period of 15 min, with the decrease of extinction being measured at λ = 339 nm. Addition of 2 μl ouabain (5 mM final concentration) or bafilomycin (Bafilomycin A1, Sigma-Aldrich) (1 μM final concentration) to the assay was used to determine the fraction of Na^+^/K^+^-ATPase or H^+^-ATPase activity from the total ATPase (TA) activity. The concentrations of inhibitors used in this enzyme assay are sufficient to fully inhibit the NKA (Morris et al., 1997) and VHA (Dröse and Altendorf, 1997), respectively. Six measurement replicates were performed for each sample (three with inhibitor dissolved in DMSO and three with DMSO). Enzyme activities were calculated by using the extinction coefficient for NADH of ε = 6.31 mM^−1^·cm^−1^ and given as micromoles of ATP consumed per gram tissue fresh mass (g_FM_) per hour.

### Preparation of mRNA

Separated gills (1–6) were homogenized in Trizol reagent (Invitrogen, Carlsbad, CA, USA) using a Tissue lyser (Quiagen). Chloroform was added to the Trizol homogenates, and total RNA was extracted from the aqueous phase and purified by addition of isopropanol. Genomic DNA contaminations were removed by DNase I (Promega, Madison, WI, USA) treatment. The mRNA for the RT-PCR was obtained using a QuickPrep Micro mRNA Purification Kit (Amersham Pharmacia, Piscataway, NJ, USA) according to the supplier protocol. Extracted mRNA concentrations were determined by spectrophotometry (ND-2000, NanoDrop Technol, Wilmington, DE), and the integrity of the mRNA was controlled by electrophoresis in RNA gels. All mRNA pellets were stored at −80°C.

### Cloning of *xtNKA* fragment

Fragments of the *X. testudinatus* Na^+^/K^+^-ATPase (NKA) and V-Type H^+^-ATPase (VHA) genes were amplified from gill tissue by means of reverse transcription followed by PCR (RT–PCR) using primers based on highly conserved regions of the NKA and VHA from the green shore crab *C. maenas*. Reverse transcription was performed as previously described (Hu et al., 2013) and the primer pair 5′-CAGTCACTTCATCCACATCA-3′ and 5′-CACATCTCCAATAGCCAGTT-3′ resulted in a 540 bp fragment of the *NKA*. PCR fragments were separated by electrophoresis in 1.5% agarose gels. Extraction, purification, and cloning of the PCR fragments from the gel was accomplished as previously described (Hu et al., 2013). Plasmids were sequenced and sequence analysis was performed using the BLASTx program (NCBI, http://blast.ncbi.nlm.nih.gov/Blast.cgi).

### Real-time quantitative PCR (qPCR)

The mRNA expressions of selected candidate genes were measured by qPCR using the Roche LightCycler® 480 System (Roche Applied Science, Mannheim, Germany). Primers for the Na^+^/K^+^-ATPase, V-type H^+^-ATPase and the reference gene arginine kinase were designed using Primer Premier software (vers. 5.0; PREMIER Biosoft International, Palo Alto, CA) and are provided in Table S2. PCR reactions were performed as previously described (Hu et al., 2013, 2014) and PCR products were subjected to a melting-curve analysis. Primer efficiencies were >96% and control reactions were performed using nuclease-free water to determine background levels. Additionally, DNAse I treated RNA samples served as a control, demonstrating that no PCR product was obtained, and thus the success of the DNase I treatment. The standard curve of each gene was in a linear range with arginine kinase (AK) that served as reference gene. The expression of this reference gene has been demonstrated to be stable in the green shore crab *C. maenas* during CO_2_ treatments (Fehsenfeld et al., 2011).

### Statistical analyses

Statistical analyses were performed using Sigma Stat 3.0 (Systat) software. Statistical differences between pH treatments within one time point were analyzed using a Student's *t*-test. The significance levels were set to *p* < 0.05^*^ and *p* < 0.01^**^.

## Results

### Extracellular acid-base status

**Figure 2:**
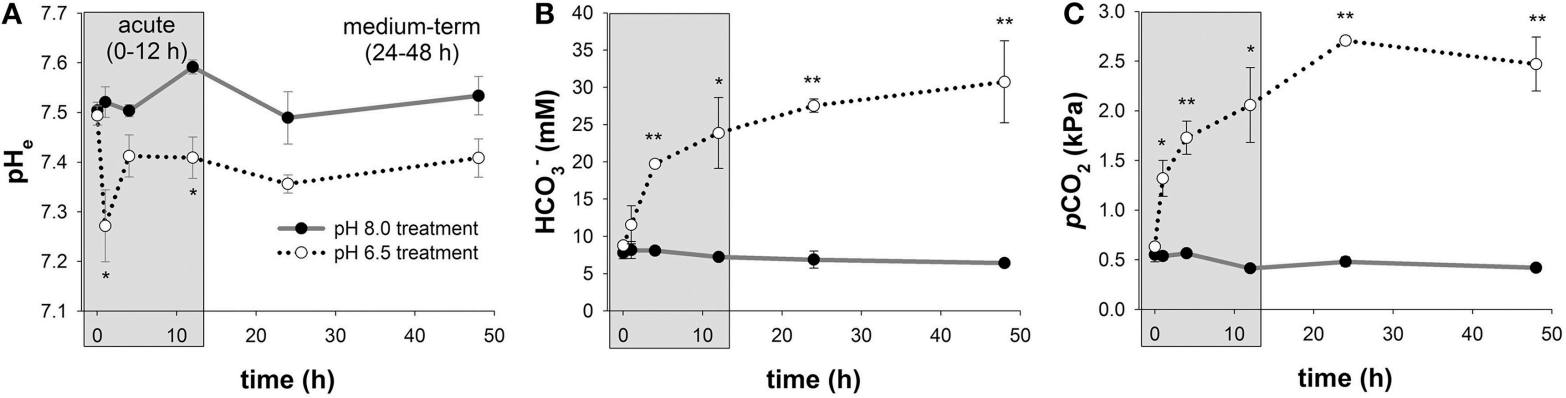
**Extracellular acid-base parameters during exposure to acidified conditions**. Time series measurements of *in vivo* extracellular pH (pHe) **(A)**, hemolymph ${\text{HCO}}_{3}^{-}$ levels **(B)**, and *p*CO_2_ **(C)** along the experimental period of 48 h. The exposure period is separated into a short-term (gray) and medium-term (white) acclimation period. Extracellular acid-base parameters are additionally presented in a Davenport diagram in Figure S1. Asterisks indicate significant differences between pH treatments (^*^p < 0.05 and ^**^p < 0.001). Bars represent mean ± SE (*n* = 4).

Mean extracellular pH (pHe) measured in hemolymph samples along the time series of 48 h ranged between pH 7.50 ± 0.02 to 7.59 ± 0.01 in control animals (Figure 2A; Figure S1). In response to acidified conditions of pH 6.5, pHe dropped (*p* < 0.05) by ~0.25 pH units compared to control animals after 1 h. pHe was partially restored after 4 h and remained stable at levels of ~0.1 pH units below control pHe. Mean blood ${\text{HCO}}_{3}^{-}$ levels were found to range from 6.4 ± 0.5 to 8.2 ± 1.1 mM in control animals along the incubation period of 48 h (Figure 2B). In response to acidified conditions of pH 6.5, hemolymph ${\text{HCO}}_{3}^{-}$ levels progressively increased from 8.8 ± 0.3 to 31 ± 6 mM during the 48 h incubation period and significant differences (*p* < 0.05 and 0.01) to control animals were observed after 4 h incubation until the end of the experiment. Hemolymph *p*CO_2_ levels ranged from 0.41 to 0.55 kPa in control animals along the incubation period of 48 h (Figure 2C). In response to acidified conditions, hemolymph *p*CO_2_ levels increased to peak values of 2.7 ± 0.3 kPa (Figure 2C).

### Regulation of branchial Na^+^/K^+^-ATPase and V-type H^+^-ATPase upon low pH exposure

**Figure 3:**
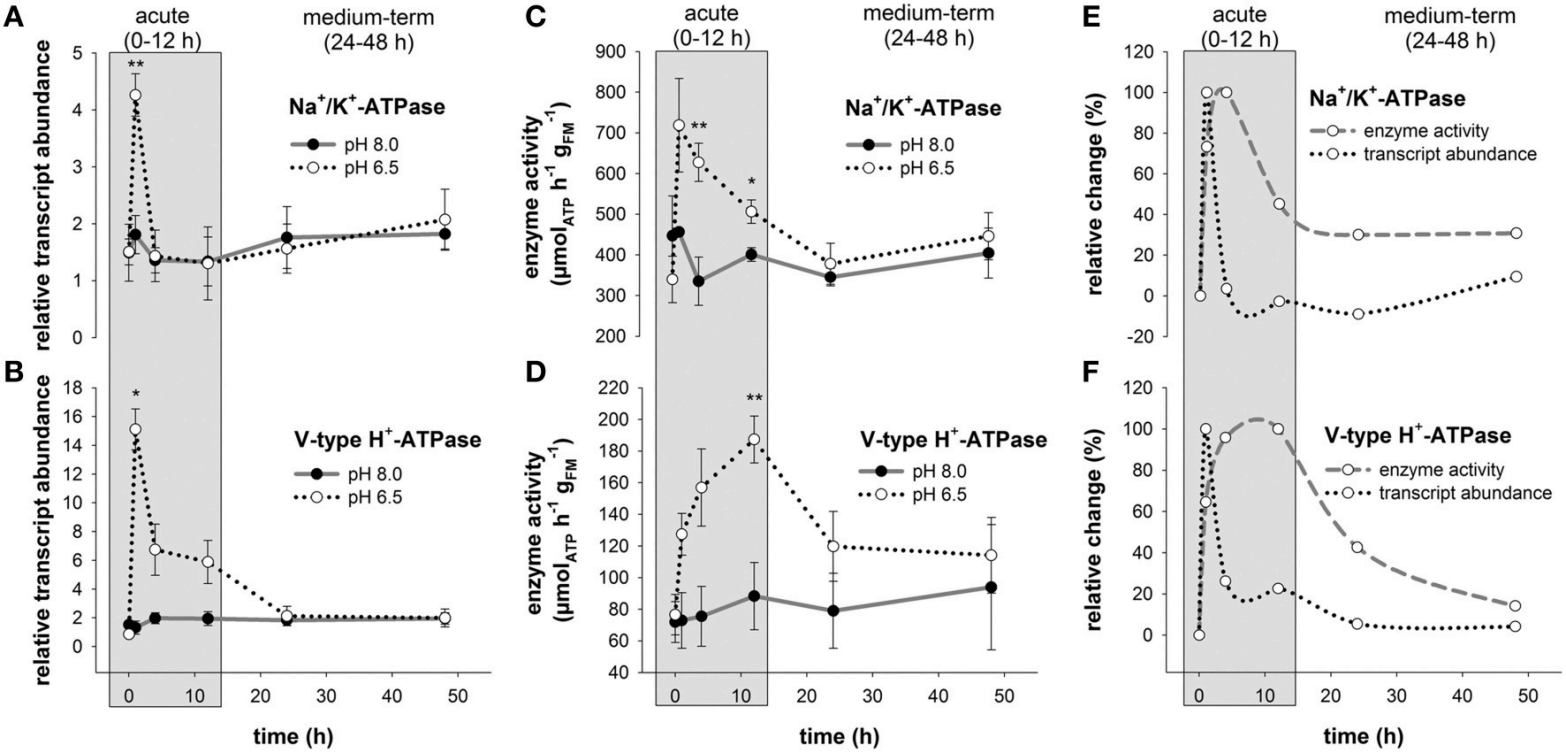
**Branchial Na^**+**^/K^**+**^-ATPase and V-type H^**+**^-ATPase transcript abundance and enzyme activities during acclimation to acidified conditions**. Relative changes in Na^+^/K^+^-ATPase (NKA) **(A)** and V-type H^+^-ATPase (VHA) **(B)** transcript levels under control and acidified conditions. Transcript levels were normalized to arginine kinase (AK) as an internal control. NKA **(C)** and VHA **(D)** enzyme activities in control and low pH treated animals along the experimental period of 48 h. Combined presentation of relative changes in NKA **(E)** and VHA **(F)** mRNA levels and enzyme activities normalized to control animals. The exposure period is separated into a short-term (gray) and medium-term (white) acclimation period. Asterisks indicate significant differences between pH treatments (^*^p < 0.05 and ^**^p < 0.001). Bars represent mean ± SE (*n* = 3–4).

Comparisons of routine mRNA levels of NKA and VHA did not reveal any significant differences between anterior (1–3) and posterior gills (4–6; Figure S2). In response to acidified conditions, mRNA levels of the NKA and VHA measured in the posterior gills (4,5,6) increased rapidly within 1 h by 2.3- and 11.4-fold, respectively (Figures 3A,B). While NKA mRNA levels returned to control levels within the acute acclimation phase of 4 h, VHA mRNA levels rapidly increased upon low pH exposure for 1 h and then decreased back to control levels within 24 h (Figures 3A,B). The increase in mRNA concentrations is paralleled by an increase in NKA and VHA enzyme activities (Figures 3C,D). Compared to control conditions where maximum NKA enzyme activities ranged from 335 ± 60 to 456 ± 9 μmol_ATP_ h^−1^ ${\text{g}}_{\text{FM}}^{-1}$, branchial NKA activities in crabs exposed to acidified conditions increased within 1 h to a maximum of 719 ± 115 μmol_ATP_ h^−1^ ${\text{g}}_{\text{FM}}^{-1}$. Along the period of 24 h, NKA activities decreased back to control levels (Figure 3C). Branchial VHA enzyme activities increased in response to acidified conditions with an activity peak after 12 h reaching 1.7-fold increased enzyme activities compared to control animals (Figure 3D). After 24 h of low pH exposure, branchial VHA activities returned back to control levels and remained slightly above control levels for the experimental period of 48 h. When comparing relative changes of NKA mRNA and activity levels along the entire experimental period a slight shift by ~3 h in peak NKA activities can be found in comparison to mRNA levels. Moreover, while NKA mRNA levels returned to control conditions after 4 h, NKA enzyme activities remain elevated by 40% to 30% along the experimental duration of 48 h (Figure 3E). A more pronounced shift between peak mRNA and enzyme activity levels of ~9 h has been observed for the branchial VHA (Figure 3F). After this acute phase, VHA enzyme activities decreased back to control levels after 48 h exposure to acidified conditions.

### Localization of Na^+^/K^+^-ATPase and V-type H^+^-ATPase in gill epithelia

**Figure 4:**
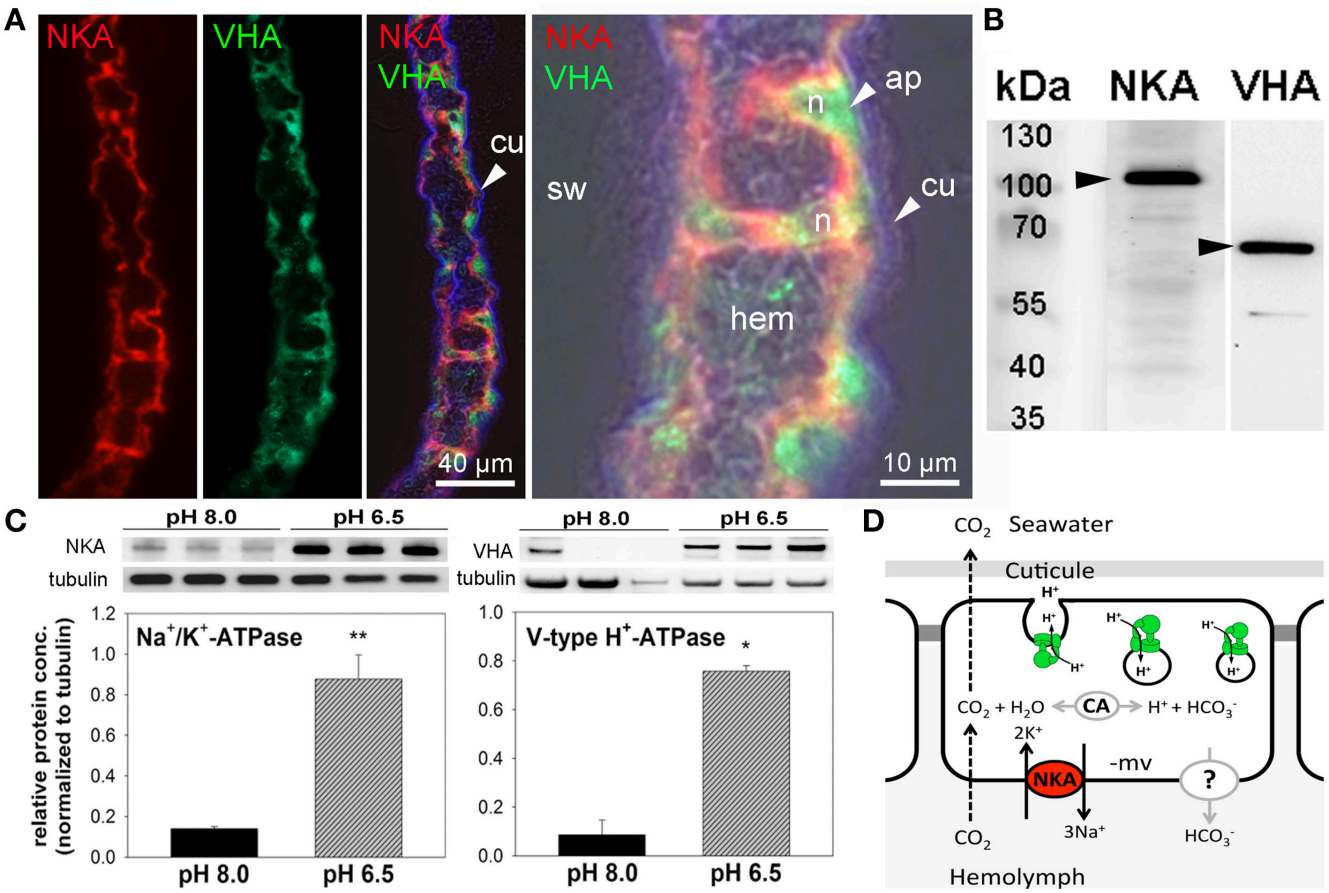
**Immuno-histochemical localization of Na^**+**^/K^**+**^-ATPase and V-type H^**+**^-ATPase in branchial epithelia**. Positive Na^+^/K^+^-ATPase (NKA) (red) immunoreactivity in basolateral membranes of the 5th gill pair of a control (pH 8) animal **(A)**. Positive immunoreactivity of the V-type H^+^-ATPase (VHA) (green) antibody is mainly located in the cytoplasm of single epithelial as well as pillar cells. Background fluorescence (blue), showing the lining of the cuticule along the gill lamellae. Larger magnification of one branchial lamella demonstrating the sub cellular localization of the NKA and VHA including a bright field overlay to visualize the histology of this tissue **(A)**. Western blot analyses using gill homogenates, indicating specific immunoreactivity of the different antibodies with proteins in the predicted size range **(B)**. Determination of NKA and VHA protein concentrations normalized to tubulin in posterior gills after 12 h exposure to pH 8.0 and 6.5 conditions (^*^p < 0.05; ^**^p < 0.01) **(C)**. Hypothetical model of acid-base regulation in epithelial cells of posterior gills in *X. testudinatus* **(D)**. NKA located in basolateral membranes energizes the import of bicarbonate via a putative basolateral ${\text{HCO}}_{3}^{-}$ transporter. CO_2_ diffuses across membranes along concentration gradients. Intracellular carbonic anhydrase (CAc) facilitates the formation of ${\text{HCO}}_{3}^{-}$ and protons. Based on the findings of the present work it is speculated that cytoplasmic VHA is involved in the acidification of vesicles and protons are exocytosed across the apical membrane. Cu, cuticula; hem, hemolymph; sw, sea water; n, nucleus; ap, apical.

Immunohistochemical analyses demonstrate the sub-cellular localization of Na^+^/K^+^-ATPase and V-type H^+^-ATPase in gill epithelia of posterior gills from control animals (Figure 4A). Using double staining, high concentrations of Na^+^/K^+^-ATPase were detected in basolateral membranes of the entire gill epithelium as well as in pillar cells spanning between the two epithelial layers (Figure 4A). V-type H^+^-ATPase was predominantly located in the cytoplasm of epithelial- and pillar- cells. In contrast to the distribution of NKA in cells of the entire gill lamella, VHA immunoreactivity is only observed in single cells, predominantly pillar cells. Negative controls performed by omitting the primary antibody, did not show any signal in posterior gill lamellae, supporting the specificity of the primary antibodies used (Figure S3). Western blot analyses demonstrate specific immunoreactivity of antibodies with proteins including Na^+^/K^+^-ATPase (≈110 kDa) and V-type H^+^-ATPase (≈70 kDa; Figure 4B). Western blot analyses of posterior gills of pH 8.0 and 6.5 treated crabs demonstrates increased NKA as well as VHA protein concentrations in low pH treated animals during the acute low pH acclimation phase (Figure 4C). NKA protein concentrations in the acute acclimation phase (12 h) are 1.4-fold higher whereas VHA protein concentrations of posterior gills are increased by 3-fold in low pH treated animals compared to pH 8.0 acclimated animals.

## Discussion

### Acid-base regulation in the vent crab *Xenograpsus testudinatus*

Substantial acid-base regulatory abilities are a characteristic of many active marine organisms including fish, cephalopods, and crustaceans (Cameron, 1978; Heisler, 1986; Pörtner et al., 1991; Melzner et al., 2009; Gutowska et al., 2010; Henry et al., 2012). In these taxa, buffering of extracellular pH (pHe) is associated with an increase in blood ${\text{HCO}}_{3}^{-}$ levels, which is a conserved and efficient mechanism to counter respiratory acidosis (Heisler, 1986; Pörtner et al., 1991; Gutowska et al., 2010; Henry et al., 2012). The present work demonstrates powerful extracellular acid-base regulatory abilities of *X. testudinatus* that are beneficial for this species to inhabit highly acidic hydrothermal vent habitats over long time scales. The hyperbolic increase in blood ${\text{HCO}}_{3}^{-}$ levels of *X. testudinatus* in response to acidified conditions is in general accordance with findings for other strong acid-base regulators (Heisler, 1986). For example, most teleosts can rapidly and fully compensate pHe during moderate to strong hypercapnia, accompanied by an increase in blood [HCO_3_^−^] in excess of 20 to 30 mM (Larsen et al., 1997; Perry et al., 2010). While cephalopods were characterized to have moderate acid-base regulatory abilities (Gutowska et al., 2010; Hu et al., 2014), most brachyuran crabs were described to be moderate to strong acid-base regulators (Cameron, 1978; Truchot, 1984; Pane and Barry, 2007; Spicer et al., 2007). Detailed investigations of extracellular acid-base parameters during hypercapnic exposure in crustaceans are restricted to a few species, including the European shore crab, *C. maenas* (Truchot, 1984), the blue crab, *Callinectes sapidus* (Cameron, 1978), *Cancer magister, Chionoecetes tanneri* (Pane and Barry, 2007), and the velvet swimming crab, *Necora puber* (Spicer et al., 2007). All species showed an initial depression of extracellular pH over the course of 4 h, which was partially restored over the next 24–48 h through an active accumulation of hemolymph ${\text{HCO}}_{3}^{-}$. Such a marked acidosis in the acute acclimation phase (0–12 h) was not observed in *X. testudinatus* exposed to pH 6.5 (1.78 kPa pCO_2_), a level greatly in excess of those experimental conditions under which *C. maenas* (0.57 kPa CO_2_; ≈pH 7.2), *C. sapidus* (1.0 kPa CO_2_; pH 7.08), *C. magister* and *C. tanneri* (1.0 kPa CO_2_; pH 7.08) were examined. As gills were demonstrated to be the major site for ion and acid-base regulation in decapod crustaceans, (Henry et al., 2012) the following paragraph focuses on the branchial mechanisms that mediate extracellular acid-base balance in the hydrothermal vent crab *X. testudinatus*.

### Branchial acid-base regulatory mechanisms

Immunohistochemical analyses demonstrate the presence of Na^+^/K^+^-ATPase and V-type H^+^-ATPase in branchial epithelia of the hydrothermal vent crab *X. testudinatus*. These primary active ion-transporters are key players for intra- and extra-cellular regulation of ion and pH homeostasis in all animals (Emery et al., 1998; Tresguerres et al., 2005; Colina et al., 2007; Horng et al., 2009). The ubiquitous NKA located in basolateral membranes creates an electrochemical gradient that fuels secondary active transporters. For example, blocking of NKA in perfused gills of the crab *Neohelice granulata* inhibited ${\text{HCO}}_{3}^{-}$ secretion and H^+^ reabsorption indicating a central role in fueling secondary active transport mechanisms relevant for acid-base regulation (Tresguerres et al., 2008).

The sub cellular localization of VHA in branchial epithelia of marine organisms seems to be less conserved compared to the NKA. For example, in most teleosts, the VHA is located in apical membranes where it is believed to mediate the direct secretion of protons (Horng et al., 2007; Tsai and Lin, 2007; Hwang et al., 2011). However, in marine species like the teleost, *Oryzias latipes* (Lin et al., 2012) and the squid *Sepioteuthis lessoniana* the VHA is located in basolateral membranes of ion-regulatory epithelia where it is involved in acid-base regulatory processes as well (Hu et al., 2013). In most crustaceans, the VHA has been demonstrated to be localized in apical membranes and/or in the cytoplasm of branchial epithelial cells (Weihrauch et al., 2001; Tsai and Lin, 2007). The strong cytosolic but weak apical abundance of the VHA in branchial cells of *X. testudinatus* is in accordance with observations made on other marine and intertidal brachyuran crabs including Ocypodid crabs, *Uca laceta*, and *Macrophthalmus* spp. as well as the brachyuran crabs, *Hemigrapsus sanguineus, H. penicillatus, Perisesarma bidens*, and *Chiromantes dehaani* (Tsai and Lin, 2007). The cytoplasmic localization of the VHA has been hypothesized to be involved in the trapping of ${\text{NH}}_{4}^{+}$ within acidified vesicles and subsequent exocytosis across the apical membrane (Weihrauch et al., 2002). As secretion of ${\text{NH}}_{4}^{+}$ also results in a net export of protons, it is likely that excretion of nitrogenous waste products and acid-base regulation are linked processes.

Gene expression analyses and enzyme activity measurements in the present study indicate that branchial NKA and VHA are important players of acid-base regulation that mediate bicarbonate accumulation as well as H^+^ secretion in this hydrothermal vent species. In fish, cephalopods and crustaceans, the compensation of an extracellular acidosis by an accumulation of ${\text{HCO}}_{3}^{-}$ is always associated with a significant net export of protons (Heisler, 1984, 1986; Cameron, 1986). As ${\text{HCO}}_{3}^{-}$ formation through the hydration of CO_2_ is always associated with the generation of H^+^, H^+^ export pathways represent an essential feature in animals that compensate an extracellular acidosis. These observations are in line with the results of the present study, demonstrating that environmental acidification stimulates expression levels of branchial VHA that is involved in the secretion of proton equivalents. Moreover, basolateral NKA has been hypothesized to energize ${\text{HCO}}_{3}^{-}$ transport in crustacean branchial epithelia (Tresguerres et al., 2008). In this context a Na^+^/${\text{HCO}}_{3}^{-}$ co-transporter (NBC) located in basolateral membranes has been proposed for the branchial epithelium of *Neohelice granulate* based on pharmacological observations (Tresguerres et al., 2008). Other studies suggested that basolateral anion exchangers are involved in the ${\text{HCO}}_{3}^{-}$ re-absorption in crustacean gills (Freire et al., 2008; Harms et al., 2014). Although the existence, function and cellular localization of ${\text{HCO}}_{3}^{-}$ transporters from the SLC 4 family are not confirmed for *X. testudinatus*, this work strongly suggests that active ${\text{HCO}}_{3}^{-}$ re-absorption significantly contributes to extracellular pH homeostasis during environmental acidification. Active ${\text{HCO}}_{3}^{-}$ transport mechanism can be considered essential for the ${\text{HCO}}_{3}^{-}$ buffering since dissolution of the carapace has been demonstrated to represent only a minor contribution to extracellular ${\text{HCO}}_{3}^{-}$ accumulation in crustaceans (Cameron, 1985). According to this information, we propose a first model for acid-base regulatory mechanisms in gill epithelia of the hydrothermal vent crab, *X. testudinatus* (Figure 4D). The NKA energizes ${\text{HCO}}_{3}^{-}$ uptake via a currently not identified basolateral ${\text{HCO}}_{3}^{-}$ transporter and cytosolic carbonic anhydrase (CA). Based on a study by Weihrauch et al. (2002) we hypothesize that also in *X. testudinatus* cytoplasmic VHA is involved in countering an acidosis by pumping H^+^ ions into vesicles that exocytose protons or proton equivalents (e.g., ${\text{NH}}_{4}^{+}$) across the apical membrane. Accordingly, during vesicle fusion at the apical membrane a certain fraction of the cellular VHA must be temporarily transferred to the apical plasma membrane as well. In order to support this hypothesis of vesicular proton excretion in this species future studies will compare the subcellular distribution of VHA-rich vesicles between control and low pH acclimated animals.

Based on the findings of the present work this model suggests that NKA seems to be involved in acid-base compensatory mechanisms by fueling additional secondary active ion-transporters. The electroneutral transport of acid-base equivalents (e.g., ${\text{HCO}}_{3}^{-}$ and H^+^) *via* anion and cation exchangers is directly connected to the transport of counter ions (e.g., Na^+^ and Cl^−^) that supports the link between acid-base and osmoregulation found in marine euryhaline decapod crustaceans (Truchot, 1981; Whiteley et al., 2001). Accordingly it is very likely that *X. testudinatus* utilizes Na^+^- and Cl^−^-dependent acid-base regulatory pathways via Na^+^/H^+^-exchangers (NHEs), and maybe also NBCs or anion exchangers that are fueled by the NKA. Using *X. testudinatus* as a model for a strong acid-base regulating crustacean further studies regarding the functional expression, enzyme abundance and subcellular localization of these secondary active transporters will provide important insights to the mechanistic connection between acid-base and osmotic regulation in decapod crustaceans.

Interestingly, the increase of the NKA and VHA on the mRNA level is only visible in the acute low pH acclimation phase, whereas enzyme activities increase with a short delay and stay elevated until the time point of 24 h. This observation reflects the time gap between mRNA expression and translation into the functional protein, which is in general accordance with findings in other eukaryotes as well as prokaryotes (Marsh et al., 2000; Glanemann et al., 2003). Although NKA and VHA enzyme activities return back to control levels after 24 h, blood ${\text{HCO}}_{3}^{-}$ levels remain increased after 48 h in the low pH treatment. This suggests, that initial accumulation of ${\text{HCO}}_{3}^{-}$ and the associated secretion of H^+^ are reflected in the up regulation of NKA and VHA mRNA and activity levels. However, after 24 h the pHe compensation process is completed and routine NKA and VHA activity levels are probably sufficient to maintain increased hemolymph ${\text{HCO}}_{3}^{-}$ levels to protect pHe homeostasis. This energy saving strategy of stabilizing hemolymph pH over long exposure times can be regarded as a key adaptation of *X. testudinatus* to occupy an ecological niche in a highly acidified hydrothermal vent habitat.

## Conclusion

**Figure 5:**
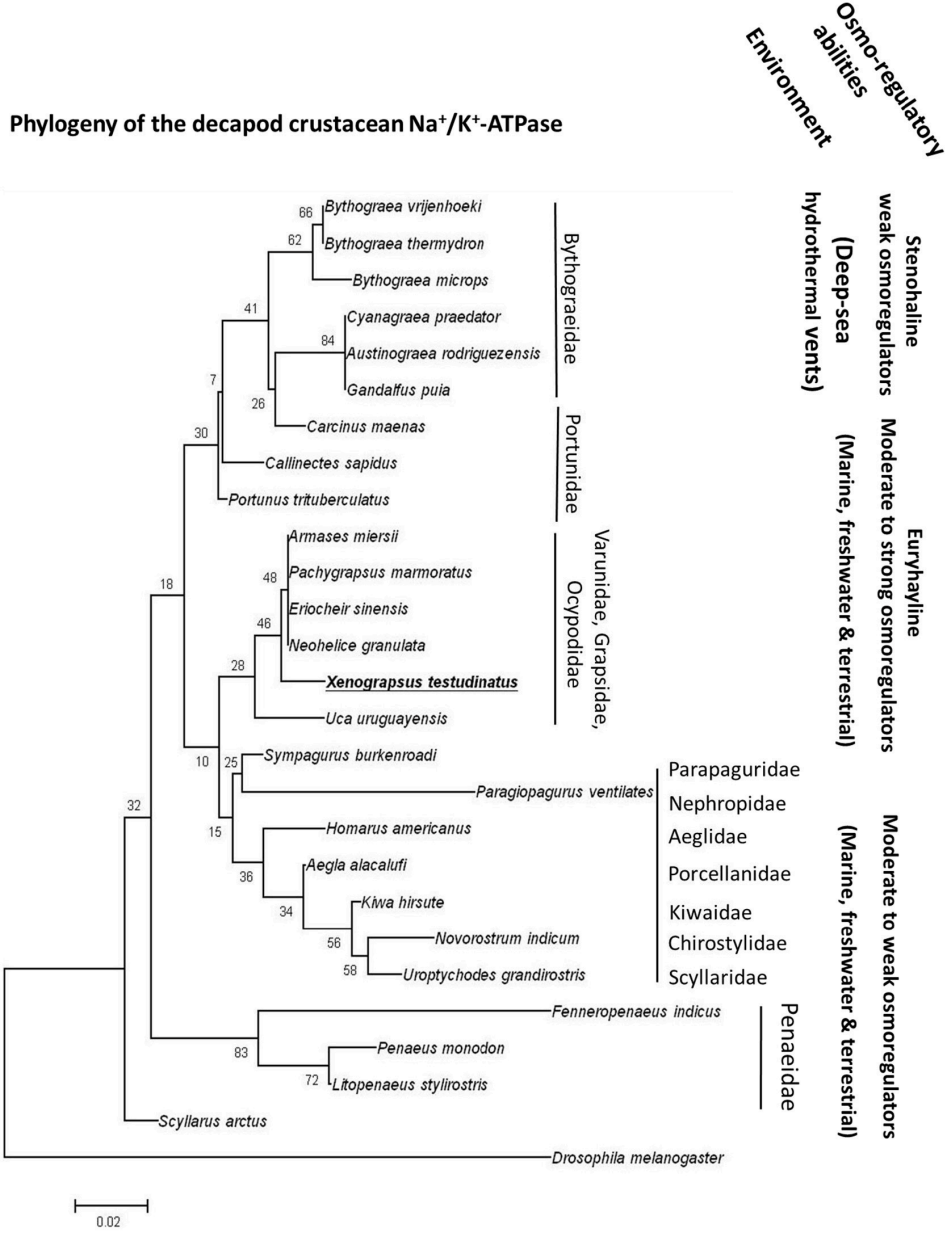
**The phylogeny of crustacean Na^**+**^/K^**+**^-ATPases**. Routed phylogenetic tree of deduced Na^+^/K^+^-ATPase amino acid sequences from different decapods crustaceans. Numbers indicate bootstrap values and accession numbers for the sequences are provided along with species names. Information regarding the habitat and osmoregulatory abilities of the different species is included in the phylogenetic tree.

Strong acid-base regulatory abilities of *X. testudinatus* can be regarded an essential feature of this species to successfully inhabit a highly acidic hydrothermal vent environment. Functional and histological results of the present study demonstrated that extracellular acid-base regulatory mechanisms in *X. testudinatus* rely on a conserved set of ion pumps that are also found in other brachyuran crabs. In accordance to phylogenetic analyses based on mitochondrial genomes (Ki et al., 2009) and morphological systematics (Ng et al., 2000), amino acid sequence comparisons of the NKA demonstrate that *X. testudinatus* is closely related to other varunid and grapsid species including *E. sinensis* and *Pachygrapsus marmoratus* which are characterized as powerful osmo-regulators (Gross, 1961; Onken, 1999; Figure 5). NKA homologies also confirm molecular systematics demonstrating that deep-sea hydrothermal vent crabs of the genus *Bythograea* spp. (Mateos et al., 2012) constitute an own phylogenetic group within the crustacea (Figure 5). Despite having weak osmo-regulating abilities, *Bythograea thermydron* (Martinez et al., 2001) may be able to increase ATPase activities to increase H^+^ efflux rates to protect from strong pH fluctuations that are a characteristic of deep-sea vent habitats (Von Damm, 1995; Tivey, 2004). However, additional studies addressing the mechanistic basis of extracellular pH regulation in crustaceans from deep-sea vent systems will be needed to support this hypothesis. Such comparative studies between deep-sea and shallow-water hydrothermal vent crabs will provide a basis for new and exciting research regarding the evolution of pH regulatory systems in crustaceans that have the ability to occupy ecological niches in highly acidic hydrothermal vent habitats.

## Author contributions

MH and YT designed and conducted experiment, analyzed the data, and compiled the main manuscript. MH conducted immunohistochemical experiments and evaluate enzyme activities. YS and MJ collected and acquired the animals and photos from open filed. YG and YT carried out the molecular cloning and expression studies. PK, GC, and JL conducted CO_2_ perturbation experiments, physiological measurements, and sample preparation. All authors reviewed and approved the manuscript.

### Conflict of interest statement

The authors declare that the research was conducted in the absence of any commercial or financial relationships that could be construed as a potential conflict of interest.
