# Supplementary material for: Strong Ion Regulatory Abilities Enable the Crab Xenograpsus testudinatus to Inhabit Highly Acidified Marine Vent Systems
Source: Front Physiol. 2016 Feb 1;7:14. doi: 10.3389/fphys.2016.00014 (PMC4734175; doi:10.3389/fphys.2016.00014)
Supplement: Supplementary file 1 [file DataSheet1.DOCX]

**Supplemental material to:**

**Strong ion regulatory abilities enable the crab *Xenograpsus testudinatus* to inhabit highly acidified vent systems**

Marian Y. Hu, Ying-Jey Guh, Yi-Ta Shao, Pou-Long Kuan, Guan-Lin Chen, Jay-Ron Lee, Ming-Shiou Jeng, Yung-Che Tseng

**
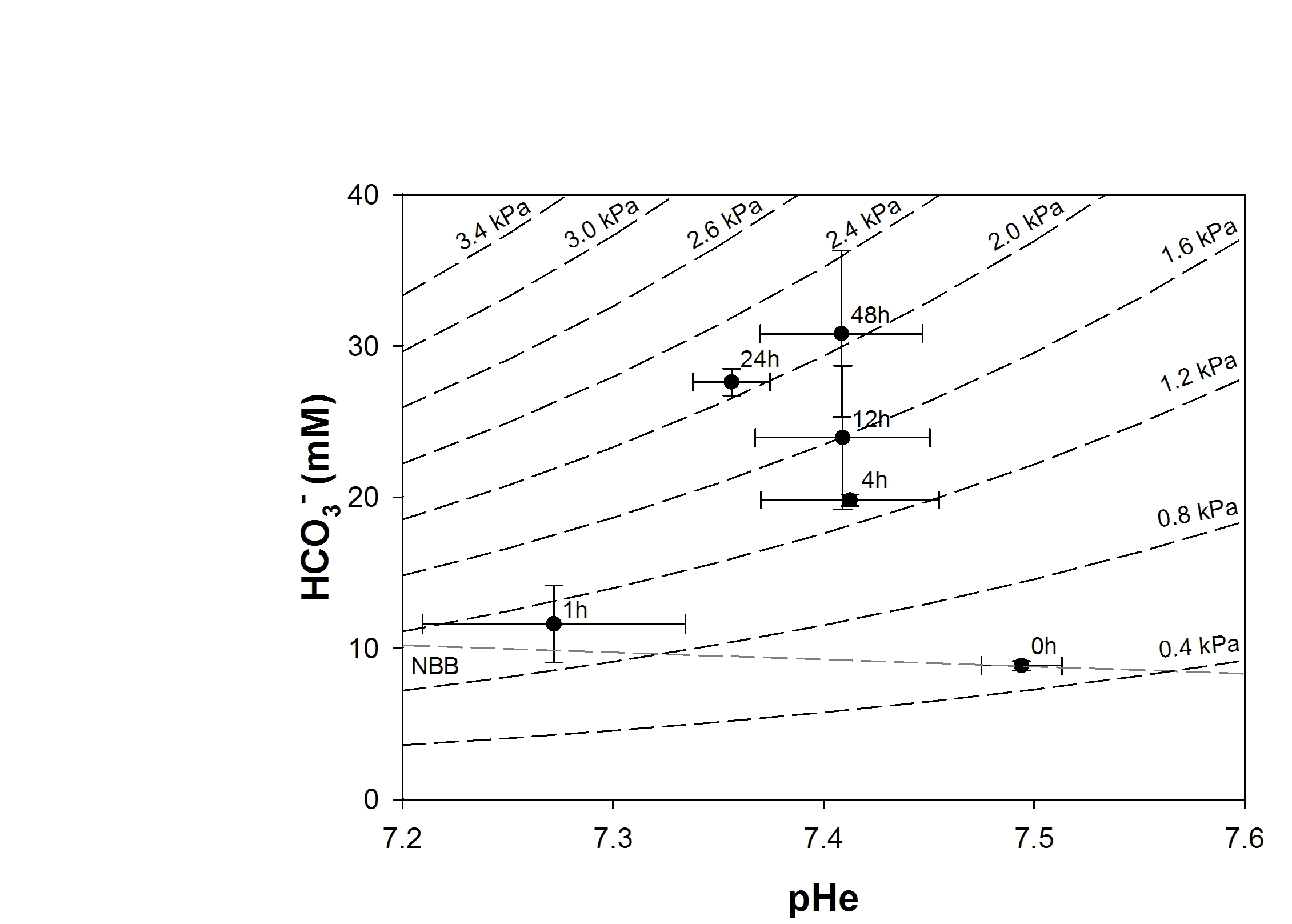
**

**Figure S1 pH-bicarbonate (Davenport) diagram demonstrating the time course of acid–base compensation in *Xenograpsus testudinatus* during exposure to acidified conditions.** The curved dashed lines are CO_2_ isopleths derived from the equation pH=6.224+log ((C*_T_*/S_CO2_) -1), where C*_T_* is total CO_2_ and Sco_2_ is physically dissolved CO_2_, both in mM. Hemolymph acid–base status was determined along the experimental period of 48 h for animals exposed to acidified (pH 6.5) conditions. Due to the limitation of hemolymph samples the non-bicarbonate buffer values were averaged from brachyuran crabs with strong acid-base regulatory abilities including *Callinectes sapidus* [^1^](#_ENREF_1) and *Cancer magister* [^2^](#_ENREF_2). Numbers in brackets indicate sampling time points. Bars represent mean ± SE (n = 4).


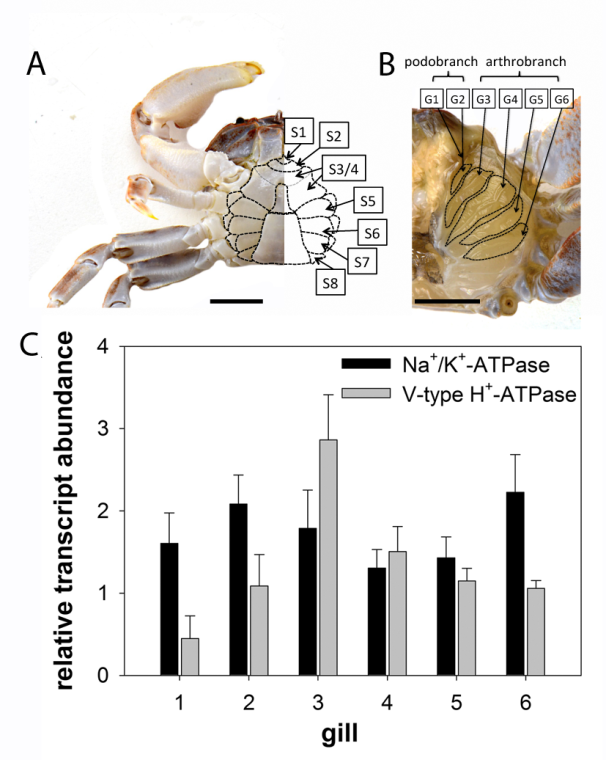


**Figure S2** **Morphology of the branchial chamber and relative transcript abundance of Na^+^/K^+^-ATPase and V-type H^+^-ATPase in various gills of *Xenograpsus testudinatus*.** X. testudinatus has eight thoracic sternal segments (S1-8) of which S3 and S4 are partly fused (A). *X. testudinatus* has six gill pairs of which gill 1 and 2 (G1+2) are podobranchs and G3 to G6 are arthrobranchs (B). Relative transcript abundance of Na^+^/K^+^-ATPase and V-type H^+^-ATPase in different gills of *X. testudinatus* (C). Transcript levels are normalized to the reference gene arginine kinase and values are expressed as mean ± SEM.


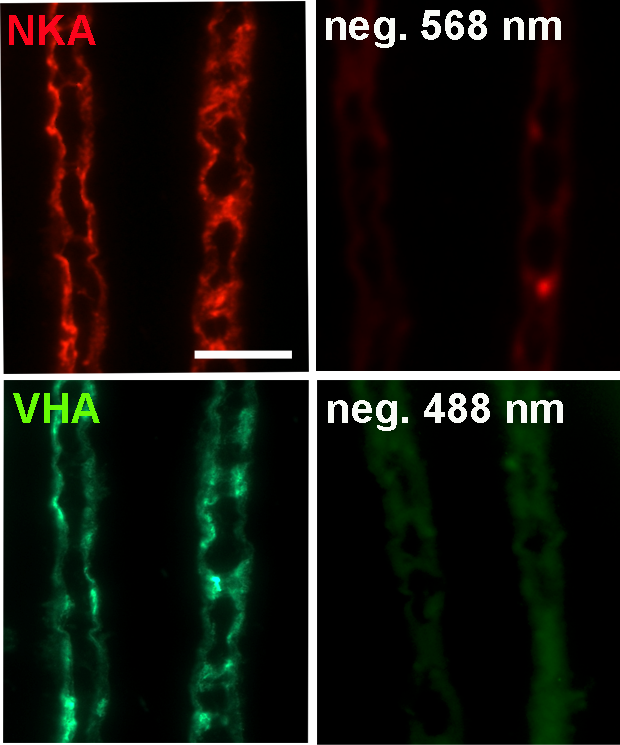


**Figure S3 Specificity control of the antibodies performed on posterior (no.5) gills of *Xenograpsus testudinatus*.** Negative controls (neg) by omitting the primary antibody Na^+^/K^+^-ATPase α (NKA) (α5) and V-type H^+^-ATPase (VHA). Positive immunoreactivity (left panels) was achieved only in the presence of both the primary and secondary antibodies. Scale bar 40 µm.

**Table S1** Seawater physiochemical conditions in experimental setups along the incubation period of 48 h

|  | **Measured** | | **Calculated** | | | |
| --- | --- | --- | --- | --- | --- | --- |
| **Treatment** | **pH_sw_** | **A_T_** | ***p*CO_2_ (kPa)** | **C_T_** | **Sal** | **Temp (°C)** |
| ̴pH 8.0 | 7.96 ± 0.05 | 2.31 ± 0.02 | 0.05 ± 0.01 | 2.05 ± 0.04 | 31 | 28 |
| ̴pH 6.5 | 6.52 ± 0.04 | 2.35 ± 0.04 | 1.78 ± 0.01 | 2.82 ± 0.09 | 31 | 28 |

**Table S2** Primer sequences used for qRT-PCR

| **Gene name** | **Abbreviation** | **Primer sequence** | **Amplicon size** | **Accession number** |
| --- | --- | --- | --- | --- |
| Na^+^/K^+^-ATPase | NKA | [F] 5'-GCCAAGTGCCGATCTGCTGGTATTA-3'  [R] 5'-GGGATGTTGAGTCTCTGGGCAATGT-3' | 143 bp | KP136453 |
| V-type H^+^-ATPase | VHA | [F] 5'-TTTACACCCTTCACCATGGCAGGA-3'  [R] 5'-ACATGGCTGACCCACTCATGTTCT-3' | 148 bp | DY656042.1 |
| **Reference gene** |  |  |  |  |
| Arginine kinase | AK | [F] 5'-CAATGGCCGACGCTGCAACTATT-3'  [R] 5'-GGTTCTCAACACCGGACTGGATCA-3' | 183 bp | AF167313.1 |

References

1 Cameron, J. N. Compensation of hypercapnic acidosis in the aquatic blue crab, *Callinectes sapidus*: The predominance of external sea water over carapace carbonate as the proton sink. *J Exp Biol* **114**, 197-206 (1985).

2 Pane, E. F. & Barry, J. P. Extracellular acid-base regulation during short-term hypercapnia is effective in a shallow-water crab, but ineffective in a deep-sea crab. *Mar Ecol Prog Ser* **334**, 1-9 (2007).
